# Development of a Recombinase Polymerase Amplification Assay for the Detection of Pathogenic *Leptospira*

**DOI:** 10.3390/ijerph110504953

**Published:** 2014-05-08

**Authors:** Ahmed Ahmed, Hans van der Linden, Rudy A. Hartskeerl

**Affiliations:** KIT Biomedical Research, WHO/FAO/OIE and National Leptospirosis Reference Centre, Royal Tropical Institute, Meibergdreef 39, Amsterdam 1105, The Netherlands; E-Mails: H.v.d.Linden@ kit.nl (H.L.); r.hartskeerl@kit.nl (R.A.H.)

**Keywords:** recombinase, polymerase, diagnosis, amplification, pathogenic, *Leptospira*, development, diagnostic, evaluation, RPA

## Abstract

Detection of leptospires based on DNA amplification techniques is essential for the early diagnosis of leptospirosis when anti-*Leptospira* antibodies are below the detection limit of most serological tests. In middle and low income countries where leptospirosis is endemic, routine implementation of real-time PCR is financially and technically challenging due to the requirement of expensive thermocycler equipment. In this study we report the development and evaluation of a novel isothermal recombinase polymerase amplification assay (RPA) for detection of pathogenic *Leptospira* based on TwistAmp chemistry. RPA enabled the detection of less than two genome copies per reaction. Retrospective evaluation revealed a high diagnostic accuracy (sensitivity and specificity of 94.7% and 97.7%, respectively) compared to culturing as the reference standard. RPA presents a powerful tool for the early diagnosis of leptospirosis in humans and in animals. Furthermore, it enables the detection of the causative agent in reservoirs and environment, and as such is a valuable adjunct to current tools for surveillance and early outbreak warning.

## 1. Introduction

Leptospirosis is a zoonotic disease with an estimated global burden of 873,000 severe annual cases and 49,000 deaths [1]. The causative agent comprises pathogenic species of the genus *Leptospira*. This bacterium circulates worldwide. It tolerates different climates and environments and can adapt to a wide range of animal hosts. Because the clinical manifestations mimic a variety of febrile infectious illnesses such as rickettsioses, dengue, and other viral haemorrhagic infections, the impact of the disease in the area where these diseases are endemic is often underrated. Because of the difficult diagnosis of leptospirosis, adequate surveillance is mostly lacking. Hence, the understanding of the transmission modes and risks and, notably, the dynamics leading to outbreaks is still in its infancy. Adequate tools for early diagnosis enabling early outbreak warning are badly needed.

Culturing from blood collected at the early stage of disease provides evidence of a leptospiral infection. However, leptospires are tedious, slow growing bacteria and it takes weeks to months for a culture to become positive. Thus, culturing as a diagnostic tool is not beneficial for the treatment of the individual patient. Nor does serology contribute to early diagnosis of leptospirosis because anti-*Leptospira* antibodies only become detectable in the late acute phase, 3–5 days after the onset of the disease. Laboratory diagnosis of leptospirosis in the early acute phase of the disease relies on molecular methods, particularly DNA amplification techniques, on blood samples [2,3]. To date, few real-time polymerase chain reactions (PCRs) have been validated and are currently in use in various laboratories. However, implementation of this technique as a routine diagnostic tool for leptospirosis is technically and financially problematic in middle and low income countries where the disease is endemic. The technique requires sophisticated expensive thermocycler equipment, subjected to regular maintenance and availability of a stable power supply. For this reason, isothermal amplification techniques avoiding the use of expensive and complicated thermocyclers in addition to the possibility of reading results by eye have been propagated as simple and affordable alternative molecular diagnostic tools [4,5,6,7,8]. However, in practice, the application of isothermal approaches such as the loop-mediated isothermal amplification (LAMP) for detection of leptospires is very limited [9]. Moreover, its diagnostic sensitivity and specificity is disputable and further intensive optimization and validation of this method is required [1]. Recently recombinase polymerase amplification (RPA) has been developed as a simple and fast isothermal amplification technique using affordable equipment. In addition to a regular DNA polymerase, this method employs a recombinase enzyme, single-stranded DNA binding proteins and homologous oligonucleotides to invade within the double-stranded target DNA, hence permitting sequence specific priming of DNA polymerase reactions without prior denaturation of template DNA. Because of the use of regular enzymes, the reaction is performed at a moderate and constant temperature (37–39 °C). Moreover, the chemistry of this technique enables both real-time readout or end-point ‘sandwich assays’, such as lateral-flow (LF) strips [10] and thus allows application in a variety of settings, ranging from sophisticated ones to point of care situations. In this study, we developed and evaluated an RPA test for detection of pathogenic *Leptospira*, using the TwistAmp Exo probe detection system for real-time readout.

## 2. Materials and Methods

### 2.1. Ethics Statement

Procedures for collecting patients’ data and use of clinical specimens for laboratory service improvement falls under the umbrella of the ‘National Coordination Infectious Disease Control’ (Landelijke Coördinatie Infectieziektebestrijding, LCI) ‘Centre for Infectious Disease Control’ (Centrum Infectieziektebestrijding, Cib) [11] which is a formal body of the Netherlands Ministry of Health and resides in the National Institute for Public Health and Environment (RIVM) in Bilthoven, The Netherlands and thus were conducted in compliance with the regulation, policies and principles of the Dutch Public Health Service Policy. The procedure includes the processing of anonymous data from patients upon receipt of a written informed consent.

### 2.2. Leptospira Strains and Others Microorganisms

Forty strains belonging to pathogenic, non-pathogenic and intermediate *Leptospira* species [12,13,14,15,16] and five other micro-organisms (Table 1) were included in this study. *Leptospira* strains were from the collection of the WHO/FAO/OIE and National Leptospirosis Reference Centre (NRL) in Amsterdam, The Netherlands. Other micro-organisms or their genomic DNA were gifts from colleagues from the Department of Biomedical Research and from other institutions.

**Table 1:** *Leptospira* strains and other microorganism used in this study.

No.	Species	Serovar	Strain	Status	Reference	Result
1	*L. alexanderi*	Manzhuang	A23	Pathogenic	[12]	+
2	*L. alexanderi*	Manhao 3	L 60	Pathogenic	[12]	+
3	*L. biflexa*	Andamana	CH 11	Non-pathogenic	[12]	−
4	*L. biflexa*	Patoc	Patoc I	Non-pathogenic	[12]	−
5	*L. borgpetersenii*	Castellonis	Castellon 3	Pathogenic	[12]	+
6	*L. borgpetersenii*	Nyanza	Kibos	Pathogenic	[12]	+
7	*L. borgpetersenii*	Hardjo-bovis	L550	Pathogenic	[12]	+
8	*L. borgpetersenii*	Ceylonica	Piyasena	Pathogenic	[12]	+
9	*L. borgpetersenii*	Mini	Sari	Pathogenic	[12]	+
10	*L. borgpetersenii*	Hardjo-Bovis	Sponselee	Pathogenic	[12]	+
11	*L. borgpetersenii*	Ballum	Mus 127	Pathogenic	[12]	+
12	*L. broomii*	Not determined	5399T	Intermediate	[13]	−
13	*L. fainei*	Hurstbridge	BUT 6T	Intermediate	[15]	−
14	*L. interrogans*	Australis	Ballico	Pathogenic	[12]	+
15	*L. interrogans*	Djasiman	Djasiman	Pathogenic	[12]	+
16	*L. interrogans*	Hardjo	Hardjoprajitno	Pathogenic	[12]	+
17	*L. interrogans*	Canicola	Hond Utrecht IV	Pathogenic	[12]	+
18	*L. interrogans*	Bratislava	Jez Bratislava	Pathogenic	[12]	+
19	*L. interrogans*	Pomona	Pomona	Pathogenic	[12]	+
20	*L. interrogans*	Copenhageni	Wijnberg	Pathogenic	[12]	+
21	*L. kirschneri*	Tsaratsovo	B 81/7	Pathogenic	[12]	+
22	*L. kirschneri*	Butembo	Butembo	Pathogenic	[12]	+
23	*L. kirschneri*	Vanderhoedeni	Kipod 179	Pathogenic	[12]	+
24	*L. kirschneri*	Grippotyphosa	Moskva V	Pathogenic	[12]	+
25	*L. kirschneri*	Cynopteri	3522 C	Pathogenic	[12]	+
26	*L. licerasiae*	Varillal	VAR 010T	Intermediate	[14]	−
27	*L. weilii*	Ranarum	ICF	Pathogenic	[16]	+
28	*L. meyeri*	Semaranga	Veldrat Semarang 173	Non-pathogenic	[12]	−
29	*L. noguchii*	Nicaragua	1011	Pathogenic	[12]	+
30	*L. noguchii*	Louisiana	LSU 1945	Pathogenic	[12]	+
31	*L. noguchii*	Argentiniensis	Peludo	Pathogenic	[12]	+
32	*L. noguchii*	Proechimys	1161 U	Pathogenic	[12]	+
33	*L. santarosai*	Balboa	735 U	Pathogenic	[12]	+
34	*L. santarosai*	Rio	Rr 5	Pathogenic	[12]	+
35	*L. santarosai*	Shermani	1342 K	Pathogenic	[12]	+
36	*L. vanthielii*	Holland	WaZHolland	Non-pathogenic	[12]	−
37	*L. weilii*	Coxi	Cox	Pathogenic	[12]	+
38	*L. weilii*	Celledoni	Celledoni	Pathogenic	[12]	+
39	*L. wolbachi*	Codice	CDC	Non-pathogenic	[12]	−
40	*L. yanagawae*	Saopaulo	Sao Paulo	Non-pathogenic	[12]	−
41	*Acinetobacter calcoaceticus*		Other microorganism			−
42	*Bacillus subtilis*		Other microorganism			−
43	*Borrelia burgdorferi*		Other microorganism			−
44	*Leptonema illini*		Other microorganism			−
45	*Treponema pallidum*		Other microorganism			−

### 2.3. Clinical Samples

This retrospective evaluation was executed on 63 clinical samples (59 serum and 4 EDTA blood) submitted to the NRL for confirmation of suspected leptospirosis All clinical specimens were collected from patients at 1 to 10 days post onset of disease (DPO). The study sample consisted of 19 samples that yielded a positive culture and 44 samples that were negative by culture. From all 19 patients who had a positive culture, leptospirosis was also confirmed by serology on paired samples, consistent with our case definition [17]. From eight of the 44 cases that scored as culture negatives, a follow up sample was received that showed negative results in the serodiagnosis. All specimens were anonymized and randomized prior to testing. Results of other diagnostic tests were unknown to the tester performing the RPA.

### 2.4. DNA Extractions

Leptospires were propagated in EMJH liquid media at 30 °C as described by Ellinghausen and McCullough [18] as modified by Johnson and Harris [19]. The concentration of bacteria was determined by counting in a Helber bacteria chamber (Weber Scientific international, West Sussex, UK) according to the standard protocol. All genomic DNAs from leptospires and other micro-organisms in culture medium and from 200 µL serum or blood were extracted, purified and eluted in 0.1 × TE buffer pH 8.0 by using the QIAamp DNA extraction kit (Qiagen, Hilden, Germany) or by using MagNA Pure Systems according to the manufacturer’s instructions (Roche Diagnostics, Mannheim, Germany). The quantity of *Leptospira* genomic DNA was estimated by measuring absorbance of DNA using the Spectrophotometer ND-1000 Nanodrop (Wilmington, DE, USA). *Leptospira interrogans* serovar Copenhageni, strain Wijnberg was used as the basic strain for the development, optimization and initial evaluation of the RPA. Based on the published genome of Copenhageni, one genome equivalent corresponds to 5.1 × 10^−15^ g DNA [20].

### 2.5. Primers and Probes Design

**Figure 1:**
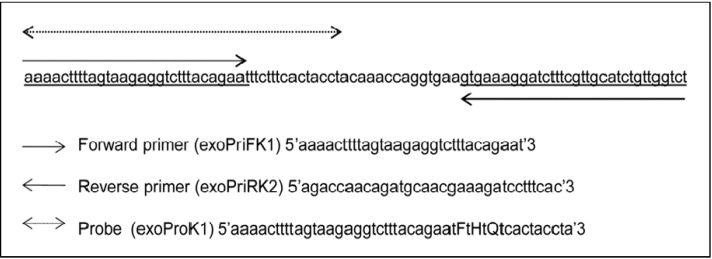
Partial *lipL32* gene sequence used as RPA amplification target. Presentation of the RPA 90 nucleotides sized amplification locus and positions of the forward (exoPriFK1) and reversed (exoPriRK2) primers and the probe (exoProK1) used in the reaction. Nucleotides are indicated by small letters. Capitals in the probe indicate the following: F = Fluorophore (FAM), H = Tetrahydrofuran (THF) spacer, Q = Black Hole Quencher-1.

Both probe (exoProK1) and forward and reverse primers (exoPriFK1 and exoPriRK2, respectively), for the recombinase polymerase amplification assay (RPA) specifically detecting pathogenic *Leptospira* were deduced from a conserved partial sequence of the gene *lipL32* from *Leptospira interrogans* serovar Copenhageni strain Fiocruz L1-130, GenBank: AE016823.1, sequence 1666423 to 1666512 (90 bp) as part of this study (Figure 1). A complete matching of these primers and probe to homologous sequence of pathogenic *Leptospira* was established by *in silico* analysis using Basic Local Alignment Search Tool (BLAST) [21]. The probe was labelled according to TwistAmp exo Probe chemistry with a fluorophore (FAM) and a Black Hole Quencher-1 (BHQ1) at its 3’ end and a tetrahydrofuran (THF) spacer in between (dTFAM-THF-dTQuencher).

### 2.6. Recombinase Polymerase Amplification: Reagents and Conditions

To perform the RPA, the TwistAmp exo kit (TwistDX, Cambridge, UK) was used. All the reagents with the exception of the rehydration buffer, magnesium acetate and the positive reaction control were included in the kit as freeze-dried pellet. The primers and the probe were added to the mix after dissolving the pellet in the 1x rehydration buffer followed by adding the target DNA template. Varying concentrations of primers, probe, magnesium acetate at different reaction temperatures varying between 37 and 41 °C, which present critical parameters, were used to select optimal RPA conditions. For optimization, the total volume of the reaction was 50 µL containing 1000 copies from *Leptospira interrogans* strain Wijnberg as DNA template. 10 µL sterile water was used instead of DNA template as a negative control. The positive control included in the kit was used to check the activity of the reagents and the performance of the reaction as recommended by the manufacturer.

The test was performed on CFX96 real-time PCR detection system (Bio-Rad). In case of an optimal amplification, the machine was programmed at constant temperature (38 °C) as follows: (i) 38 °C for 4 min as incubation period followed by mixing and a spin down step (ii) then incubation at 38 °C for 2 s followed by (iii) fluorescence detection. The last two steps (ii) and (iii) were repeated 120 times. The total reaction time was 25 minutes.

### 2.7. Determination of Analytical and Diagnostic Sensitivity and Specificity

The analytical sensitivity and the performance of the assay was monitored as described previously [20], using genomic DNA isolated from *L. interrogans* serovar Copenhageni strain Wijnberg. Briefly, 10-fold serial dilutions of genomic DNA were used as template to perform the test and to construct an amplification standard curve. The last 10-fold dilutions still giving a positive signal were subjected to subsequent 2-fold serial dilutions. We set the end-point at the dilution in which the assay detects the target in at least 95% of the replicates. The amplification performance of the RPA was compared with that of two previously published real-time PCRs, namely SYBR green PCR targeting *secY* [20] and TaqMan PCR targeting *lipL32* [22], both performed as described by the authors. The analytical specificity was examined using 45 *Leptospira* strains and other microorganism as listed in Table 1. The diagnostic sensitivity and specificity and the confidence intervals were calculated using the 63 clinical samples according to standard literature [23,24,25]. All samples used in this study were tested in duplicate.

## 3. Results

### 3.1. Optimal Reaction Conditions and RPA Analytical Sensitivity and Specificity

A reaction temperature of 38 °C and concentrations of 420 nM for each the primer, 60 nM for the probe and 16.8 mM magnesium acetate were found to provide an optimal performance of the RPA. Applying this condition, appropriate exponential amplification was achieved and the detection limit of the assay (LOD) was estimated at ≤10 fg or ≤2 genome equivalents based on the standard curve constructed from serial dilutions of genomic DNA. This compares well with the LOD of the two real-time PCRs included in the evaluation, which also allowed detection of about two genome copies (results not shown). RPA gave positive results for all pathogenic *Leptospira*, and a negative result for all saprophytic and intermediate species as well as for all other pathogenic microorganisms included in the evaluation, consistent with the specificity of the test for pathogenic *Leptospira* spp. as deduced from *in silico* analysis (Table 1).

### 3.2. The Diagnostic Sensitivity and Specificity

63 clinical samples were enrolled in this study to calculate the diagnostic sensitivity and specificity. Based on results from culturing as the reference test, RPA had diagnostic sensitivity (DSe) and specificity (DSp) of 94.7% (95% CI, 71.9 to 99.7%) and 97.7% (95% CI, 86.5 to 99.9%), respectively. Among 19 positive culture patient samples, one gave negative result when tested with RPA assay. In contrast, one culture negative sample gave a positive result in RPA.

## 4. Discussion

In this study, we report for the first time the development and evaluation of an RPA assay for the detection of pathogenic *Leptospira* [10]. The gene *lipL32* was used as the amplification target for RPA. *lipL32* codes for a major lipoprotein located in the outer membrane. The gene is confined to pathogenic *Leptospira* spp. and its overall sequence is well conserved [26]. A high analytical specificity was obtained by selecting an amplification locus of 90 nucleotides within this gene for deducing primers and a probe that shared 100% sequence homology with all available sequences from strains of pathogenic *Leptospira* spp. The *in silico* specificity of the assay was confirmed by application of RPA on a panel of bacteria, including saprophytic and intermediate *Leptospira* spp., confirming that amplification was restricted to pathogenic leptospiral strains. These pathogenic strains included strain ICF that previously has been re-positioned from the saprophytic species, *L. meyeri*, to the pathogenic *Leptospira* clade. [12,16,20,27,28,29,30,31,32,33], once again confirming its pathogenic status.

In this study the analytical sensitivity was estimated based on counting the number of the bacteria in culture prior to DNA extraction and a subsequent determination of the concentration of the DNA by spectrophotometry. The detection limit, established at < 2 genome copies, compared well with that of two validated real-time PCRs, one based on SYBR Green technology and the other one on TaqMan chemistry [20,22]. This substantiates a similar technical performance of this much simpler RPA compared to the sophisticated PCR formats.

To determine the diagnostic accuracy of RPA, culturing was used as the reference test. Culturing provides proof of infection and previous reports suggest that culturing as a reference test is superior to the standard microscopic agglutination test (MAT) [17,34]. Our evaluation revealed a very satisfying level of diagnostic accuracy for RPA with a DSe and DSp of 94.7% and 97.7%, respectively. One sample, consisting of EDTA blood gave a positive result by RPA however it was negative by culturing. From this patient, no follow-up sample, required for serological confirmation, was obtained. However, the diagnostic SYBR Green PCR on the single sample gave an indeterminate outcome, suggesting that leptospiral DNA might have been present in the sample at a low concentration. Furthermore, a positive RPA associated with a negative culture result can well be explained both by stochastic effects as well as by taking into account that EDTA is deleterious to the viability of leptospires [35]. The possibility of false positive result by carryover contamination is not obvious, since this format of RPA reaction is performed in a closed system that evades the risk of cross-over. A stochastic cause is also a plausible explanation for the one sample that was negative by RPA and positive by culture. The culture was found to belong to a pathogenic species *L. interrogans* serovar Hebdomadis (data not shown). Thus, the possibility of a negative RPA result because of an infection with an intermediate species is excluded. This is not surprisingly as isolation of leptospires belonging to intermediate species generally is associated with asymptomatic infection or mild disease [13,14,15]. Additionally, the retrospective nature of this study implies that RPA has been executed on samples that have been stored for months to years, not always under conditions that optimally preserve the integrity of the leptospiral DNA. RPA amplifies a much shorter DNA segment than is usually targeted by PCR. Hence, it is less susceptible to detrimental effects on the quality and quantity of the amplification target during storage. However, damage of the target DNA leading to a reduced apparent diagnostic sensitivity cannot be fully ruled out in this evaluation. Indeed, this specific sample had been stored for 2 years and a consequent false negative outcome of RPA presents a likely explanation. This is further substantiated by the fact that leptospirosis was also confirmed by serology on a paired sample using the standard microscopic agglutination test.

RPA with a real-time readout offers marked advantages above other previously described amplification assays for *Leptospira* detection, including isothermal ones. The technique is more rapid than any other amplification method, generating a result within 25 min and the reaction is performed at an ambient constant temperature. Consequently, it evades the need for an expensive and sophisticated thermocycler. Basically, it only requires a simple battery driven handheld fluorometer in point-of-care and field setting.

RPA is less sensitive to inhibitors than PCR [10] and is capable of amplifying DNA from a variety of samples from humans, animals and the environment. The test allows multiplexing and thus has a potential use for differential diagnosis of several clinically similar diseases [36] or allows detailing of the infecting *Leptospira* at the species and even strain level. These are important features for adequate surveillance. In principle, this real-time RPA approach generates digital data that can be linked to the Global System for Mobile Communications (GSM) network and, hence, might be helpful in creating diagnostic data clouds, accessible through internet by various stakeholders (clinicians, patients, epidemiology sections at national reference centers and/or Ministries of Health and Agriculture) through dedicated passwords. Simplification is possible by using the endpoint amplification read-out through the lateral flow platform [10], which shows a promising but not yet optimal performance (data not shown). The lateral flow presents a point-of-care device, which can be labelled with a Quick Response (QR) code linked to clinically and epidemiologically relevant data. The image of the test result and code could then be transmitted by SMART phone. Alternatively, sophisticated but more reliable lab-on-a-chip approaches are possible, for example by supplying the device with a microchip that becomes active by hybridization to the detection strip. Thus RPA presents a potentially powerful and innovative tool for surveillance and early outbreak detection that could contribute to a better understanding and prediction of outbreaks. Our study shows that RPA may present a highly valuable and attractive alternative to early diagnosis by currently available molecular amplification methods. However, our study has been performed retrospectively and comprised a relative small sample of cases, hence affecting the statistical validity of the data. Since leptospirosis is a relatively rare disease in the Netherlands, a prospective study including statistically relevant numbers will take several years of execution. To overcome this drawback, we aim at a further prospective evaluation of the diagnostic accuracy of this RPA in settings where leptospirosis is more prevalent.

## 5. Conclusions

In this study for the first time, we describe and evaluate an RPA assay for the detection of pathogenic *Leptospira* in clinical samples. The analytical and diagnostic sensitivity and specificity of the assay were satisfactory. RPA is an isothermal reaction which can be performed with simple equipment therefore RPA is particularly suitable in point-of-care and field setting. The method is very fast, less sensitive to amplification inhibitors and enables both real-time readout or end-point detection. Our test has capability to detect the pathogen in reservoir animals and in a variety of environment samples. Hence, the *Leptospira* RPA could contribute to the effort toward better understanding the dynamic of the infection during outbreaks.
